# Supplementary material for: Chp1 is a dedicated chaperone at the ribosome that safeguards eEF1A biogenesis
Source: Nat Commun. 2024 Feb 15;15:1382. doi: 10.1038/s41467-024-45645-w (PMC10869706; doi:10.1038/s41467-024-45645-w)
Supplement: Supplementary file 1 — Supplementary Information [file 41467_2024_45645_MOESM1_ESM.pdf]

# Supplementary Information

## Chp1 is a dedicated chaperone at the ribosome that safeguards eEF1A biogenesis

Melania Minoia[1†], Jany Quintana-Cordero[1†], Katharina Jetzinger [1,2], Ilgin Eser Kotan[2], Kathryn Jane Turnbull[3,4], Michela Ciccarelli[1], Anna E. Masser[1], Dorina Liebers[2], Eloïse Gouarin[1], Marius Czech[1], Vasili Hauryliuk[5,6], Bernd Bukau[2], Günter Kramer[2], Claes Andréasson[1*]

[1]Department of Molecular Biosciences, The Wenner-Gren Institute, Stockholm University, Stockholm, Sweden

[2]Center for Molecular Biology of the University of Heidelberg (ZMBH), DKFZ-ZMBH Alliance, Heidelberg, Germany

[3] Department of Clinical Microbiology, Rigshospitalet, 2200 Copenhagen, Denmark

[4]Department of Molecular Biology, Laboratory for Molecular Infection Medicine Sweden, Umeå Centre for Microbial Research, Science for Life Laboratory, Umeå University, Umeå, Sweden

[5] Science for Life Laboratory, Department of Experimental Medical Science, Lund University, Lund, Sweden.

[6]University of Tartu, Institute of Technology, 50411 Tartu, Estonia

†Contributed equally

[*] Corresponding author. Email: claes.andreasson@su.se

This file includes:

**Supplementary Figures 1 to 8**
**Supplementary Tables 1 to 2**

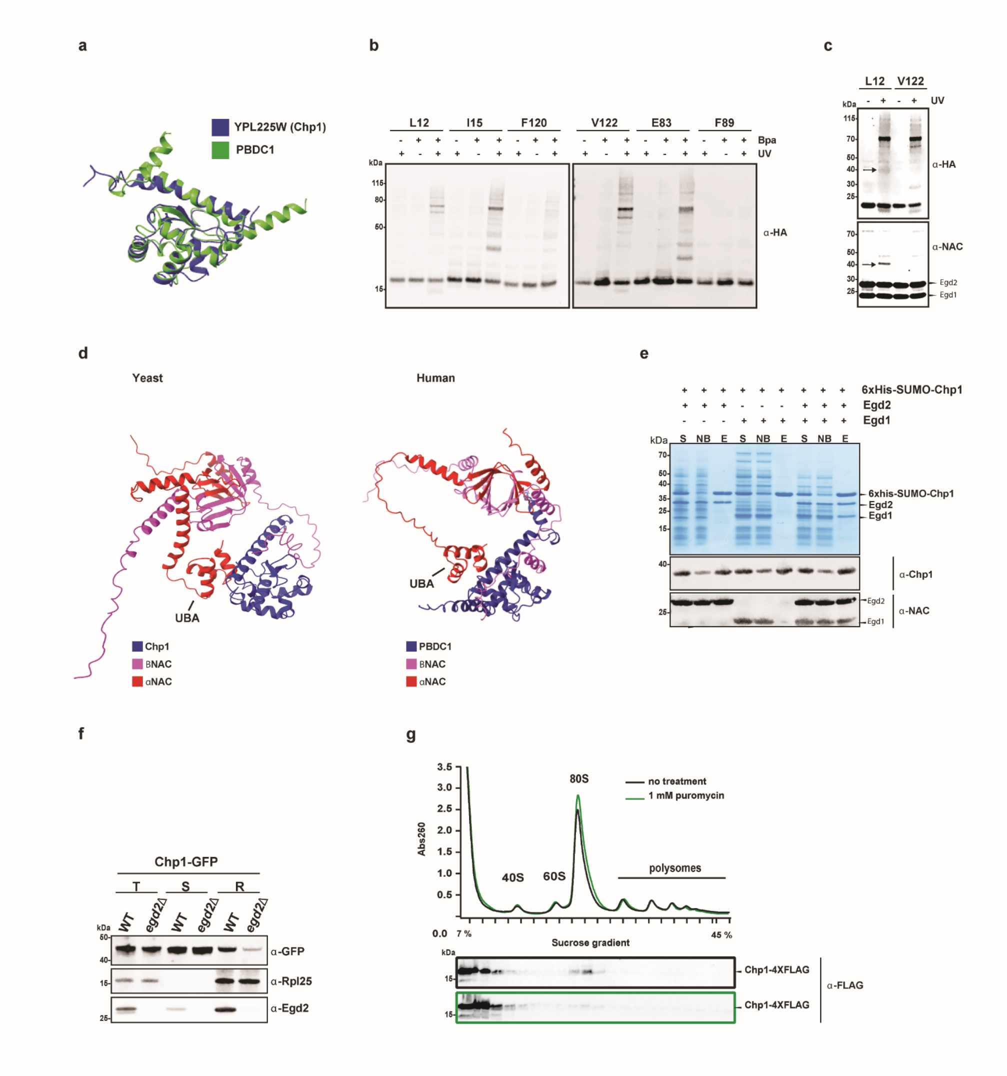


**Supplementary Fig. 1: Chp1 is a highly conserved protein that forms a complex with NAC and RNC complexes. a** Overlap of 3D-structures of yeast Chp1 in blue (PDB ID. 2JYN) and the ColabFold model of the human ortholog PBDC1 (Uniprot accession: Q08971, amino acids 13-172,) in green. **b** Photo-crosslinking (+UV) of yeast cells grown at 30 °C expressing Chp1-HA with Bpa (+Bpa) incorporated at amino acid positions 12, 15, 120, 122, 83 or 89. Proteins were visualized by western blot analysis. Experiment was performed three times. **c** Photo-crosslinking (+UV) of yeast cells grown at 30 °C expressing Chp1-HA-myc-8xHis with Bpa incorporated at amino acid positions 12 or 122. Proteins were visualized by western blot analysis. Experiment was performed three times. **d** Ribbon diagram of ColabFold models showing the predicted complex between Chp1 and NAC from yeast and humans. Chp1 and PBDC1 in blue, βNAC in violet (amino acids 32-191 of the human protein are shown) and αNAC in red (amino acids 64-215 of the human protein are shown). The UBA domain of αNAC in the yeast and human proteins is marked. Note that the heterodimerization domain structurally determined by crystallography is arranged in a head-to-head manner that generates a 12-stranded β-barrel-like heterodimer with six strands in each of the two major β-sheets, while the models display 6:4 and 5:4 β-strand symmetry for the human and yeast proteins, respectively. **e** Soluble (S), non-bound, (NB), and eluted (E) fractions of 6xHis-Sumo-Chp1 purification from *E. coli* coexpressing Egd1 and Egd2. Experiment was performed three times. **f** Total soluble cell lysate (T) of the indicated yeast strains was separated into ribosomal (R) and soluble (S) fractions and the localization of Chp1-GFP and Rpl25 among the different fractions was analyzed by western blot. Experiment was performed three times. **g** Polysome profile analysis of yeast cells expressing Chp1-4xFLAG after treatment with 1 mM of puromycin (green) or in the absence of treatment (black). Co-migration of Chp1-4xFLAG with ribosomes along the polysome profile fractions was analyzed by western blot. Experiment was performed two times.

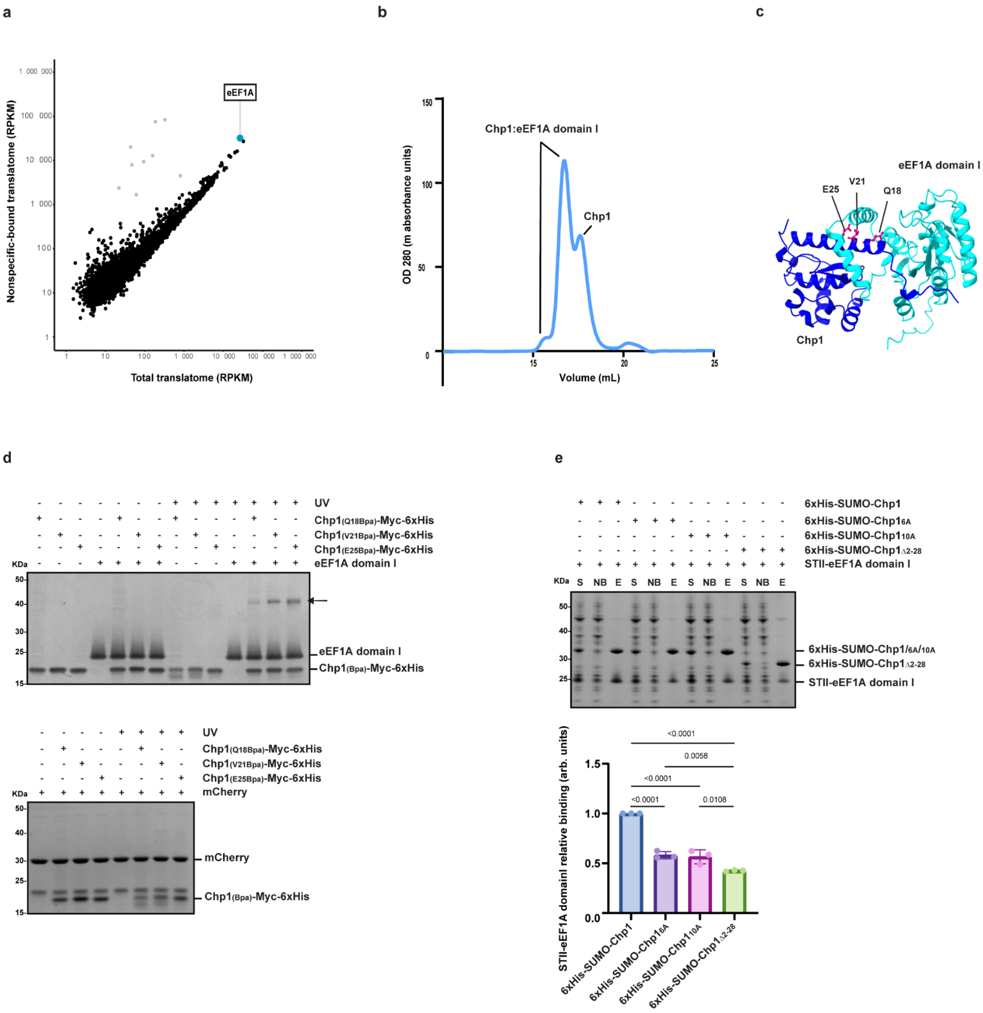

**Supplementary Fig. 2: Chp1 binds the GTPase domain of eEF1A. a** Gene expression levels in the nonspecific-bound translatome and the total translatome in reads per kilobase million (RPKM) obtained via SeRP from an untagged control yeast strain. Merged reads for the genes *TEF1* and *TEF2* which both encode eEF1A are shown in blue. Genes overrepresented in the Chp1-bound translatome but that also appeared overrepresented in the nonspecific matrix-bound translatome are shown in light grey. **b** SEC analysis of a mix of Chp1 and eEF1A domain I copurified from an *E. coli* system of recombinant expression. Peaks corresponding to monomeric Chp1 and Chp1- eEF1A domain I complex are marked. **c** Ribbon diagram of the ColabFold model of the complex between Chp1 and eEF1A domain I. Single residues of Chp1 that have been substituted by Bpa (Q18, V21, E25) for UV-inducible crosslinking studies are marked and shown in pink ball and sticks (Chp1 - dark blue, eEF1A domain I – cyan). **d** *In vitro* photo-crosslinking (+UV) between purified Chp1-Myc-6xHis with Bpa incorporated at amino acid positions 18, 21 or 25 and purified domain I of eEF1A. The protein mCherry was used to control for unspecific crosslinking. Proteins were analyzed by SDS-PAGE and Coomassie Brilliant Blue staining. The crosslinking product between Chp1-Myc-6xHis and eEF1A domain I is marked with an arrow. Experiment was performed three times. **e** STII-eEF1A domain I was coexpressed in *E. coli* with either 6xHis-SUMO-Chp1, 6xHis-SUMO-$Chp1_{6A}$ (L12A, D14A, I15A, F19A, V21A, E25), 6xHis-SUMO-$Chp1_{10A}$ (T3A, F4A, E7A, T8A, L12A, D14A, I15A, F19A, V21A, E25A) or 6xHis-SUMO-$Chp1_{\Delta 2\text{-}28}$. Soluble fractions of the cell lysates (S) were subjected to IMAC purification and the binding of eEF1A domain I to the different mutant variants of Chp1 was analyzed by SDS-PAGE and Coomassie Brilliant Blue staining. NB (non-bound) and E (eluted) fractions from the IMAC purification. Quantifications represent means ±SD, n=3 independent experiments using one-way ANOVA.

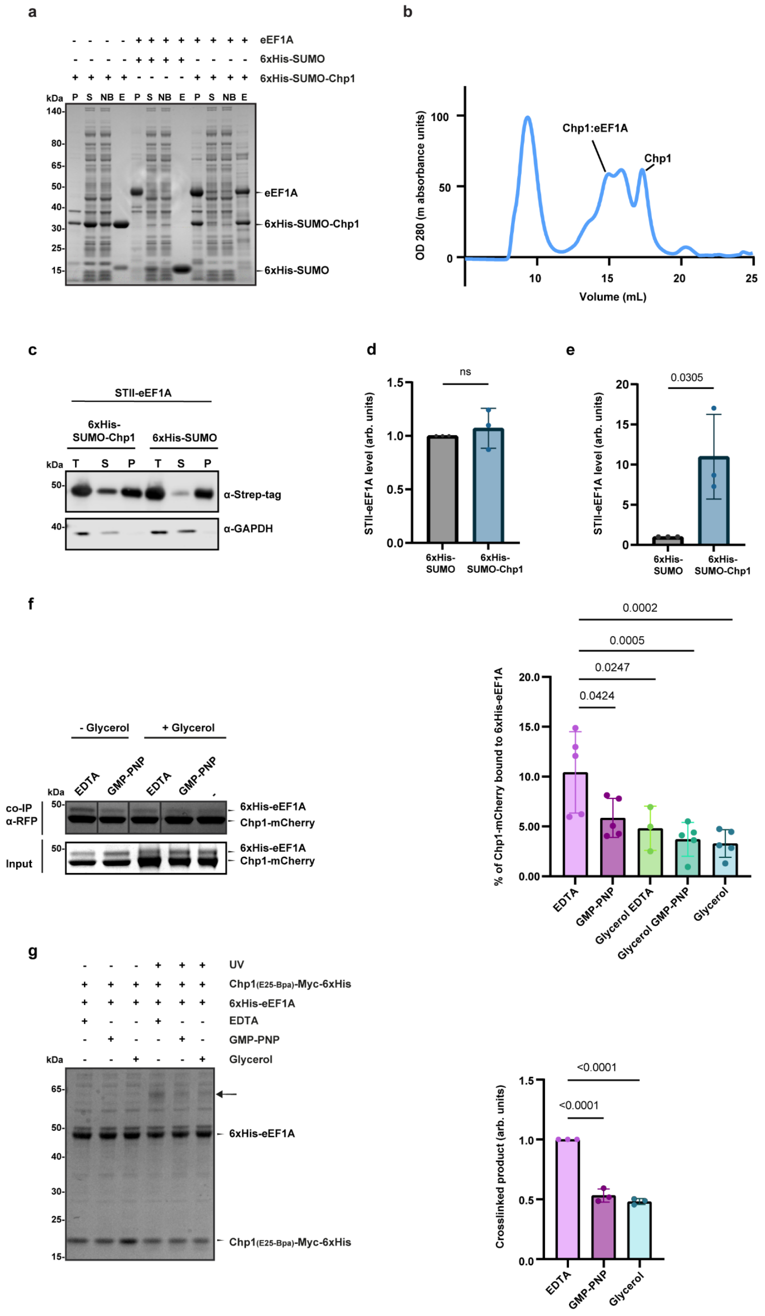


**Supplementary Fig. 3: Chp1 binds misfolded eEF1A. a** eEF1A was co-expressed with 6xHis-SUMO-Chp1 or 6xHis-SUMO in *E. coli*. Total cell lysate (T) was separated into pellet (P) and soluble (S) fractions and the S fraction was subjected to IMAC purification. NB (non-bound) and E (eluted) fractions from the IMAC purification. All fractions were analyzed by SDS-PAGE followed by Coomassie Brilliant Blue staining. Experiment was performed three times. **b** SEC analysis of a mix of Chp1 and eEF1A obtained in the E fraction of the copurification in (**a**). **c** Total cell lysate (T) from *E. coli* cells coexpressing STII-eEF1A and 6xHis-SUMO-Chp1 or 6xHis-SUMO was separated into soluble (S) and pellet (P) and the level of STII-eEF1A in the different fractions was analyzed by western blot. **d** Quantification of the level of recombinant STII-eEF1A in the T fraction from (**c**) (means ±SD, n=3 independent experiments, two-tailed t test). **e** Quantification of the level of recombinant STII-eEF1A in the S fraction from (**c**) (means ±SD, n=3 independent experiments, two-tailed t test). **f** *In vitro* anti-RFP co-IP of Chp1-mCherry and 6xHis-eEF1A (preincubated with either EDTA (10 mM), GMP-PNP (1 mM), glycerol (25% v/v), glycerol (25% v/v) and EDTA (10 mM) or glycerol (25% v/v) and GMP-PNP (1 mM). Lines between samples indicate cropping from the same gel to remove irrelevant lanes. Quantifications represent means ±SD, n=3 (Glycerol EDTA n=3) independent experiments, one-way ANOVA. **g** *In vitro* photo-crosslinking (+UV) between Chp1-Myc-6xHis with Bpa incorporated at amino acid position 25 and 6xHis-eEF1A (preincubated with either EDTA (10 mM), GMP-PNP (1 mM) or glycerol (25% v/v) ). Proteins were analyzed by SDS-PAGE and Coomassie Brilliant Blue staining. The crosslinking product between Chp1-Myc-6xHis and 6xHis-eEF1A is marked with an arrow. Quantifications represent means ±SD, n=3 independent experiments, one-way ANOVA.

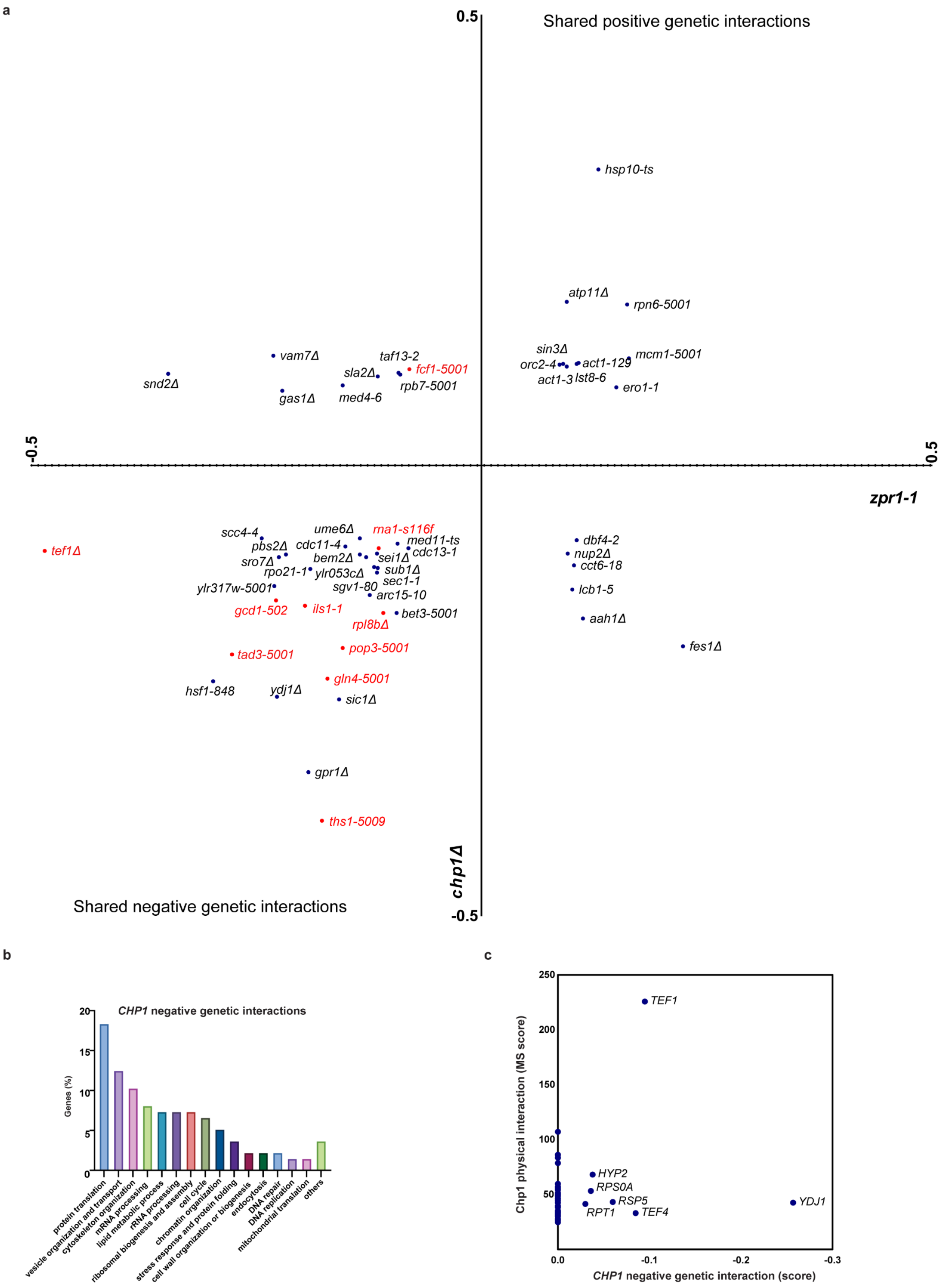


**Supplementary Fig. 4: *chp1* genetic interactions. a** Analysis of data from TheCellMap showing significant genetic interactions that are common to *chp1*Δ and *zpr1-1* (intermediate cutoff: score < -0.08 and > 0.08 for negative and positive genetic interactions respectively, p-value < 0.05). Genes involved in translation are labeled in red. **b** Analysis of functional categories of *CHP1* negative genetic interactions. **c** Analysis of *CHP1* negative genetic interactions with genes encoding proteins identified by MS as Chp1 physical interactors involved in translation and protein quality control. Genes encoding Chp1 physical interactors that show negative genetic interaction with *CHP1* are labeled.

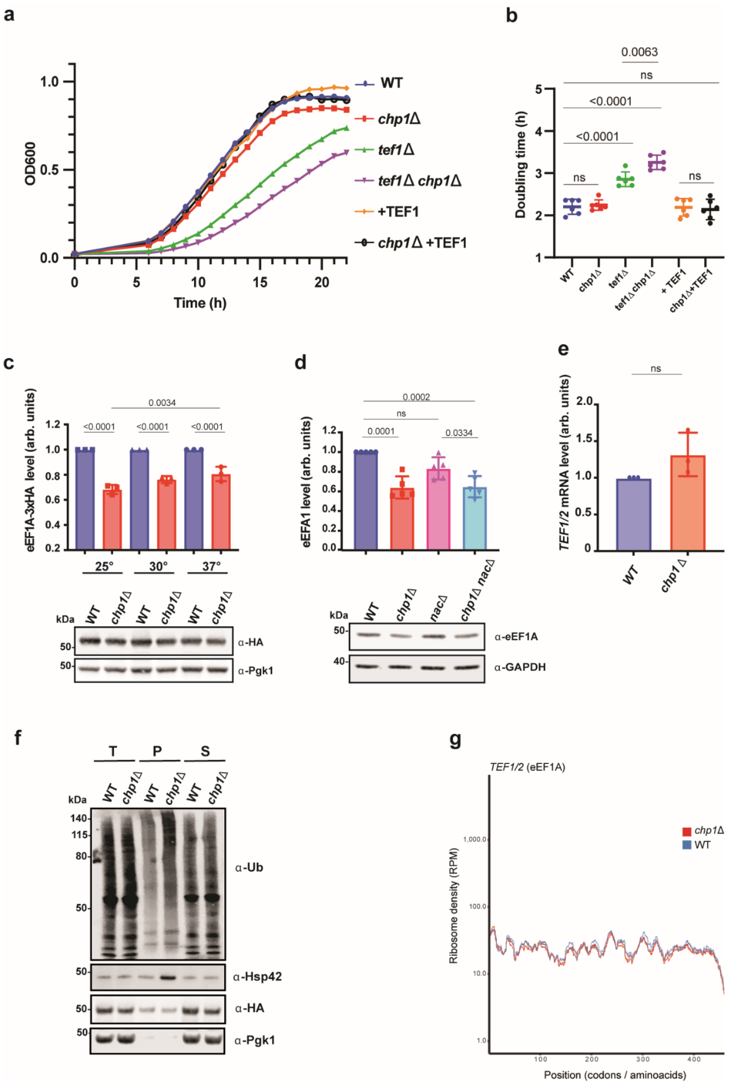


**Supplementary Fig. 5: eEF1A-linked phenotypes of *chp1*Δ.** **a** Growth curves of WT, *chp1*Δ, *tef1*Δ*, tef1*Δ *chp1*Δ*, +TEF1, chp1*Δ *+TEF1* strains grown at 30 °C in SC medium. Data represent means (n=4 independent experiments). **b** Doubling times of strain in (**a**) at the exponential growth. Data represent means ±SD (n=6 independent experiments, one-way ANOVA). **c** Expression level of eEF1A (Tef1-3xHA) in WT and *chp1*Δ cells at 25 °C, 30 °C and 37 °C (means ±SD, n=3 independent experiments, one-way ANOVA). **d** Expression level of eEF1A in WT, *chp1*Δ , *nac*Δ and *chp1*Δ *nac*Δ cells (means ±SD, n=5 independent experiments, one-way ANOVA). **e** Analysis of *TEF1* and *TEF2* mRNA levels in WT and *chp1*Δ yeast strains normalized to *TAF10* mRNA level at 30 °C. Data represent means ±SD (n=3 independent experiments, two-tailed t test). **f** Protein aggregation analysis of extracts from WT and *chp1*Δ cells expressing Tef1-3xHA. Total protein lysates (T) were separated into pellet (P) and soluble (S) fractions through centrifugation. All fractions were analyzed by western blot. Experiment was performed three times. **g** Ribosome density in reads per million (RPM) along the *TEF1*/*2* transcripts positions obtained via ribosome profiling (RP) of WT and *chp1*Δ cells.

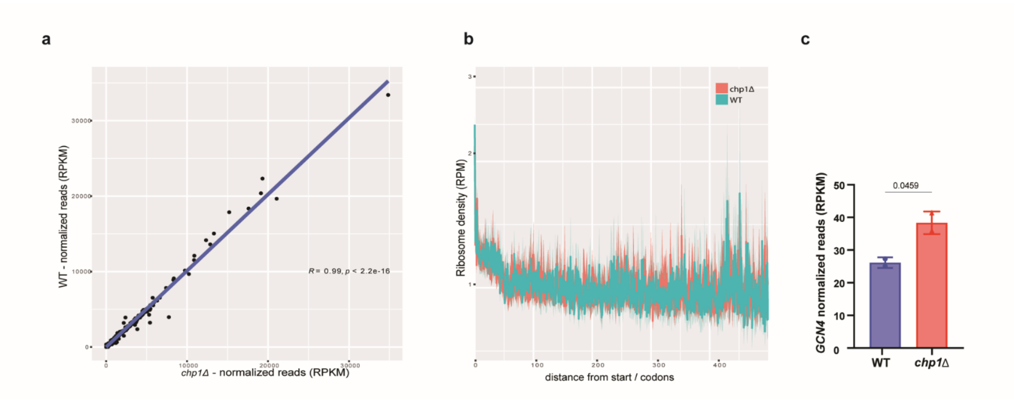


**Supplementary Fig. 6: Global translation in *chp1*Δ cells. a** Gene expression (RPKM) in WT and *chp1*Δ cells obtained by ribosome profiling. **b** Average ribosome density on all coding sequences (metagene profile) of *chp1*Δ and WT cells. **c** *GCN4* expression (RPKM) in WT and *chp1*Δ cells obtained via ribosome profiling. Data represent means ±SD (n=2 independent experiments, two-tailed t test).

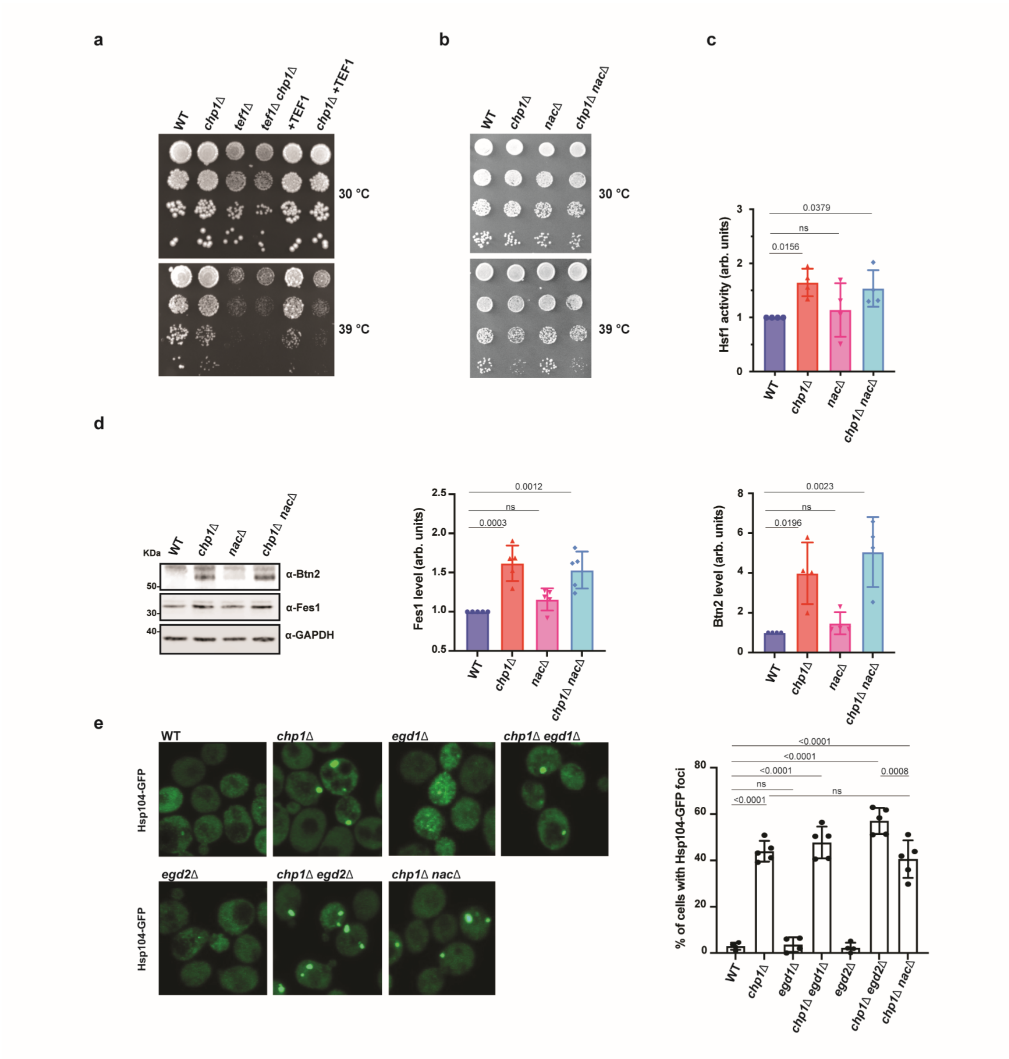


**Supplementary Fig. 7: Cells lacking Chp1 display strong proteostasis defects linked to eEF1A expression. a** Growth assay of WT, *chp1*Δ, *tef1*Δ, *tef1*Δ *chp1*Δ, *+TEF1* and *chp1*Δ *+TEF1* yeast strains at 30 and 39 °C on YPD plates after 3 days. **b** Growth assay of WT, *chp1*Δ, *nac*Δ and *chp1*Δ *nac*Δ yeast strains at 30 and 39 °C on SC plates after 3 days. **c** Hsf1 activity in WT, *chp1*Δ, *nac*Δ, *chp1*Δ *nac*Δ yeast strains determined by the **P**$_{CYC1}$-HSE-yNluc bioluminescent reporter. Data represent means ±SD (n=4 independent experiments, one-way ANOVA). **d** Expression levels of Fes1 and Btn2 in WT, *chp1*Δ, *nac*Δ, *chp1*Δ *nac*Δ yeast strains determined by western blot analysis. Quantifications represent means ±SD (n=4 independent experiments, one-way ANOVA). **e** Fluorescent micrographs of Hsp104-GFP in cells with the indicated genotype. Quantification of the fraction of cells with Hsp104-GFP foci. Data represent means ±SD (n=5 independent experiments, one-way ANOVA).

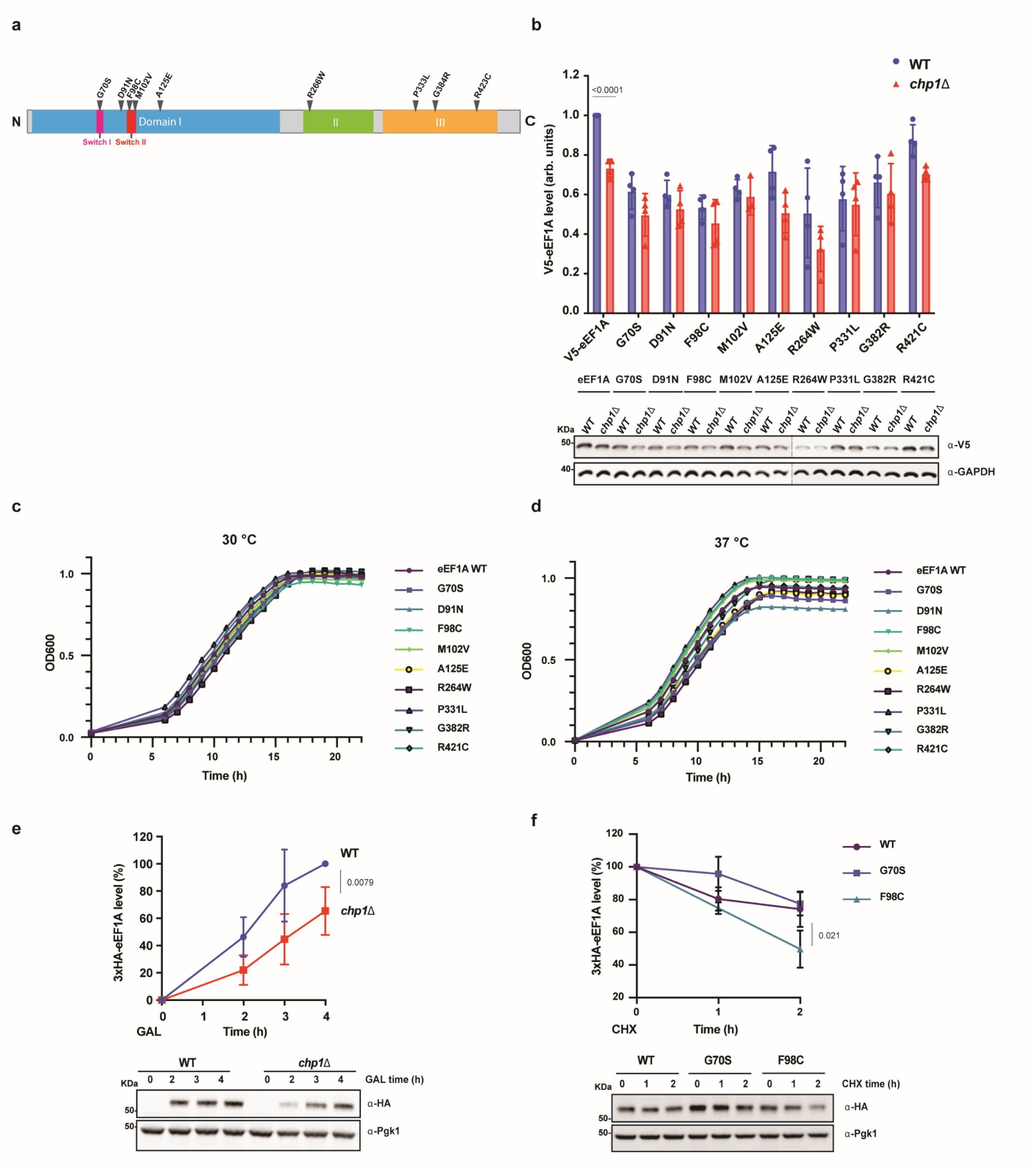


**Supplementary Fig. 8: Pathogenic *EEF1A2* mutations are destabilizing and impact on eEF1A expression levels.** **a** Human eEF1A2 pathogenic variants associated with neurodevelopmental disorders marked on a schematic representation of the human eEF1A2. Domains I, II and III are marked as well as the Switch I and II regions of the GTPase domain. **b** Expression levels of control V5-eEF1A (V5-TEF2) and the pathogenic variants (G70S, D91N, F98C, M102V, A125E, R264W, P331L, G382R and R421C) in WT and *chp1*Δ cells. Quantifications represent means ±SD (n=4 independent experiments). **c** Growth curves at 30 °C in SC medium of yeast cells expressing WT V5-eEF1A (V5-Tef2) or each of the pathogenic variants (G70S, D91N, F98C, M102V, A125E, R264W, P331L, G382R and R421C). Data represent means (n=4 independent experiments). **d** As in (**c**) but at 37 °C. **e** Time course of *de novo* expression of 3xHA-eEF1A from the *GAL1* promoter in WT and *chp1*Δ cells after galactose (GAL) induction. Quantifications represent means ±SD (n=4 independent experiments, two-tailed t test). **f** Time course of the stability of control 3xHA-eEF1A and G70S and F98C derivatives in WT cells after 3 hours of galactose induction following translational arrest with 0.1 mg/mL CHX. Quantifications represent means ±SD (n=4 independent experiments, two-tailed t test).

**Supplementary Table 1.** Yeast strains.

| Strain | Genotype | Source |
|---|---|---|
| AMY58 | MATa his3Δ1 leu2Δ0 ura3Δ0 trp1Δ::kanMX chp1Δ::hphNT1 | This work |
| BY4741 | MATa his3Δ1 leu2Δ0 met15Δ0 ura3Δ0 | Brachmann et al., 1998 |
| CAY1015 | MATa his3Δ1 leu2Δ0 ura3Δ0 | Gowda et al., 2013 |
| CAY1255 | MATa his3D1 leu2D0 met15D0 ura3D0 HSP104-eGFP-his3MX6 | EUROSCARF |
| CAY1363 | MATa his3Δ1 leu2Δ0 met15Δ0 ura3Δ0 chp1Δ::kanMX | EUROSCARF |
| CAY1366 | MATa his3Δ1 leu2Δ0 ura3Δ0 chp1Δ::kanMX | This work |
| CAY1367 | MATa his3D1 leu2D0 met15D0 ura3D0 HSP104-eGFP-his3MX6 chp1Δ::kanMX | This work |
| CAY1371 | MATa his3Δ1 leu2Δ0 ura3Δ0 CHP1-sfGFP::kanMX | This work |
| JQY5 | MATa his3Δ1 leu2Δ0 ura3Δ0 CHP1-4×FLAG-kanMX | This work |
| JQY7 | MATa leu2Δ0 ura3Δ0 chp1Δ::kanMX his3Δ1::[HIS3; TEF1] | This work |
| JQY9 | MATa leu2Δ0 ura3Δ0 his3Δ1::[HIS3; TEF1] | This work |
| JQY10 | MATa his3D1 leu2D0 ura3D0 URA3-**P**TDH3-6×His-TEF2 | This work |
| JQY11 | MATa his3D1 leu2D0 ura3D0 chp1Δ::kanMX URA3-**P**TDH3-6xHis-TEF2 | This work |
| MMY09 | MATa his3Δ1 leu2Δ0 ura3Δ0 can1Δ::**P**CHP1-sfGFP-kanMX | This work |
| MMY105 | MATa his3Δ1 leu2Δ0 ura3Δ0 natNT2-$P_{GAL1}$-3xHA-TEF1 | This work |
| MMY108 | MATa his3Δ1 leu2Δ0 ura3Δ0 chp1Δ::kanMX natNT2-$P_{GAL1}$-3xHA-TEF1 | This work |
| MMY111 | MATa his3Δ1 leu2Δ0 ura3Δ0 tef1Δ::hphNT1 | This work |
| MMY12 | MATa his3Δ1 leu2Δ0 met15Δ0 ura3Δ0 HSP104-eGFP::hisMX6 chp1Δ::kanMX egd2Δ::natNT2 | This work |
| MMY13 | MATa his3Δ1 leu2Δ0 ura3Δ0 CHP1-sfGFP-kanMX egd2Δ::natNT2 | This work |
| MMY130 | MATa his3Δ1 leu2Δ0 met15Δ0 ura3Δ0 HSP104-yeGFP::kanMX | Verena Kohler |
| MMY131 | MATa his3Δ1 leu2Δ0 met15Δ0 ura3Δ0 HSP104-yeGFP::kanMX chp1Δ::natNT2 | This work |
| MMY132 | MATa his3Δ1 leu2Δ0 met15Δ0 ura3Δ0 HSP104-yeGFP::kanMX tef1Δ::hphNT1 | This work |
| MMY134 | MATa his3Δ1 leu2Δ0 ura3Δ0 tef1Δ::hphNT1 chp1Δ::natNT2 | This work |
| MMY139 | MATa his3Δ1 leu2Δ0 met15Δ0 ura3Δ0 HSP104-yeGFP::kanMX his3Δ1:: [HIS3; TEF1] | This work |
| MMY14 | MATa his3Δ1 leu2Δ0 met15Δ0 ura3Δ0 HSP104-eGFP-hisMX6 egd2Δ::natNT2 | This work |
| MMY141 | MATa his3Δ1 leu2Δ0 met15Δ0 ura3Δ0 HSP104-yeGFP::kanMX chp1Δ::natNT2 his3Δ1::[HIS3; TEF1] | This work |
| MMY149 | MATa his3Δ1 leu2Δ0 met15Δ0 ura3Δ0 Hsp104-yeGFP::kanMX chp1Δ::natNT2 tef1Δ::hphNT1 | This work |
| MMY17 | MATa his3Δ1 leu2Δ0 met15Δ0 ura3Δ0 HSP104-eGFP-hisMX6 chp1Δ::kanMX egd1Δ::hphNT1 | This work |
| MMY18 | MATa his3Δ1 leu2Δ0 ura3Δ0 CHP1-sfGFP-kanMX egd1Δ::hphNT1 | This work |
| MMY18 | MATa his3Δ1 leu2Δ0 ura3Δ0 CHP1-sfGFP-kanMX egd1Δ::hphNT1 | This work |
| MMY24 | MATa his3Δ1 leu2Δ0 met15Δ0 ura3Δ0 HSP104-eGFP::hisMX6 chp1Δ::kanMX egd2Δ::natNT2 egd1Δ::hphNT1 | This work |
| MMY66 | MATa his3Δ1 leu2Δ0 met15Δ0 ura3Δ0 egd2Δ::natNT2 btt1Δ::hphNT1 egd1Δ::hisMX6 | This work |
| MMY69 | MATa his3Δ1 leu2Δ0 met15Δ0 ura3Δ0 chp1Δ::kanMX egd2Δ::natNT2 btt1Δ::hphNT1 egd1Δ::hisMX6 | This work |
| MMY89 | MATa his3Δ1 leu2Δ0 ura3Δ0 CHP1-sfGFP-kanMX egd1Δ::hphNT1 btt1Δ::hisMX6 egd2Δ::natNT2 | This work |
| MMY92 | MATa his3Δ1 leu2Δ0 ura3Δ0 TEF1-3×HA-hphNT1 | This work |
| MMY94 | MATa his3Δ1 leu2Δ0 ura3Δ0 chp1Δ::kanMX TEF1-3xHA-hphNT1 | This work |
| MMY150 | MATa his3Δ1 leu2Δ0 ura3Δ0 lys2Δ::PGAL1-HA-NatNT2-TEF2 | This work |
| MMY152 | MATa his3Δ1 leu2Δ0 ura3Δ0 chp1Δ::kanMX lys2Δ::PGAL1-HA-NatNT2-TEF2 | This work |
| MMY154 | MATa his3Δ1 leu2Δ0 ura3Δ0 lys2Δ::PGAL1-HA-NatNT2-TEF2 F98C | This work |
| MMY155 | MATa his3Δ1 leu2Δ0 ura3Δ0 chp1Δ::kanMX lys2Δ::PGAL1-HA-NatNT2-TEF2 F98C | This work |
| MMY157 | MATa his3Δ1 leu2Δ0 ura3Δ0 lys2Δ::PGAL1-HA-NatNT2-TEF2 G70S | This work |
| MMY158 | MATa his3Δ1 leu2Δ0 ura3Δ0 chp1Δ::kanMX lys2Δ::PGAL1-HA-NatNT2-TEF2 G70S | This work |

**Supplementary Table 2.** Plasmids.

| Plasmid | Description | Type | Source |
|---|---|---|---|
| ECYRS-BpA | BPa system *TRP1* AmpR | 2 micron | Chen et al,. 2007 |
| pAM17 | ***P***$_{CYC1(HSE)}$-*yNlucPEST URA3* AmpR KanR | CEN/ARS | Masser et al,. 2016 |
| pCA1000 | **P**$_{T7}$-lacO-6×His-SUMO-Chp1 lacI KanR | *E. coli* | This work |
| pCA1034 | **P** $_{T7}$-lacO-6×His-SUMO-Chp1-SD-StrepTag II-Egd2 lacI KanR | *E. coli* | This work |
| pCA1038 | **P** $_{T7}$-lacO-6×His-SUMO-Chp1-SD-StrepTag II-Egd2-SD-Egd1 lacI KanR | *E. coli* | This work |
| pCA1048 | **P** $_{T7}$-lacO-6×His-SUMO-StrepTag II-Egd2-SD-Egd1 lacI KanR | *E. coli* | This work |
| pJQ7 | **P**$_{T7}$-lacO-6×His-SUMO-Chp1-mCherry lacI KanR | *E. coli* | This work |
| pJQ8 | **P**$_{T7}$-lacO-6×His-SUMO-mCherry lacI KanR | *E. coli* | This work |
| pJQ12 | **P** $_{T7}$-lacO-6×His-SUMO-Chp1-SD-StrepTag II-*TEF2* lacI KanR | *E. coli* | This work |
| pJQ14 | **P** $_{T7}$-lacO-6×His-SUMO-SD-StrepTag II-*TEF2* lacI KanR | *E. coli* | This work |
| pJQ15 | **P** $_{T7}$-lacO-6×His-SUMO-Chp1-SD-StrepTag II-*TEF2*$^{(1\text{-}238)}$ lacI KanR | *E. coli* | This work |
| pJQ17 | **P** $_{T7}$-lacO-6×His-SUMO-SD-StrepTag II-*TEF2*$^{(1\text{-}238)}$ lacI KanR | *E. coli* | This work |
| pJQ20 | **P** $_{T7}$-lacO-6×His-SUMO-Chp1-SD-StrepTag II-*TEF2*$^{(1\text{-}70)}$ lacI KanR | *E. coli* | This work |
| pJQ21 | **P** $_{T7}$-lacO-6×His-SUMO-SD-StrepTag II-*TEF2*$^{(1\text{-}70)}$ lacI KanR | *E. coli* | This work |
| pJQ22 | **P**$_{T7}$-lacO-6×His-SUMO-*TEF2*$^{(1\text{-}238)}$ lacI KanR | *E. coli* | This work |
| pJQ24 | HIS3 YIP *TEF1* AmpR | *YIP* | This work |
| pJQ26 | **P**$_{T7}$-lacO-Chp1$^{(Q18Bpa)}$-Myc-6×His KanR | *E. coli* | This work |
| pJQ27 | **P**$_{T7}$-lacO-Chp1$^{(V21Bpa)}$-Myc-6×His KanR | *E. coli* | This work |
| pJQ28 | **P**$_{T7}$-lacO-Chp1$^{(E25Bpa)}$-Myc-6×His KanR | *E. coli* | This work |
| pJQ31 | **P** $_{T7}$-lacO-6×His-SUMO-StrepTag II-Egd2$^{(\Delta UBA)\text{-}}$SD-Egd1 lacI KanR | *E. coli* | This work |
| pJQ32 | **P** $_{T7}$-lacO-6×His-SUMO-Chp1$_{10A}$ $^{(T3A,\ F4A,\ E7A,\ T8A,\ L12A,\ D14A,\ I15A,\ F19A,\ V21A,\ E25A)}$-SD-StrepTag II-*TEF2*$^{(1\text{-}238)}$ lacI KanR | *E. coli* | This work |
| pJQ33 | **P** $_{T7}$-lacO-6×His-SUMO- Chp1$_{6A}$ $^{(L12A,\ D14A,\ I15A,\ F19A,\ V21A,\ E25)}$-SD-StrepTag II-*TEF2*$^{(1\text{-}238)}$ lacI KanR | *E. coli* | This work |
| pJQ34 | **P** $_{T7}$-lacO-6×His-SUMO-6xHis-SUMO-Chp1$^{\Delta 2\text{-}28}$-SD-StrepTag II-*TEF2*$^{(1\text{-}238)}$ lacI KanR | | |
| p416GPD V5-TEF2 | **P**$_{GPD}$-*V5-TEF2 URA3* AmpR | CEN/ARS | Carvill et al., 2020 |
| Do. A125E | **P**$_{GPD}$-*V5-tef2-A125E URA3* AmpR | CEN/ARS | Carvill et al., 2020 |
| Do. D91N | **P**$_{GPD}$-*V5-tef2-D91N URA3* AmpR | CEN/ARS | Carvill et al., 2020 |
| Do. F98C | **P**$_{GPD}$-*V5-tef2-F98C URA3* AmpR | CEN/ARS | Carvill et al., 2020 |
| Do. G382R | **P**$_{GPD}$-*V5--tef2-G382R URA3* AmpR | CEN/ARS | Carvill et al., 2020 |
| Do. G70S | **P**$_{GPD}$-*V5-tef2-G70S URA3* AmpR | CEN/ARS | Carvill et al., 2020 |
| Do. M102V | **P**$_{GPD}$-*V5-tef2-M102V URA3* AmpR | CEN/ARS | Carvill et al., 2020 |
| Do. P331L | **P**$_{GPD}$-*V5-tef2-P331L URA3* AmpR | CEN/ARS | Carvill et al., 2020 |
| Do. R264W | **P**$_{GPD}$-*V5-tef2-R264W URA3* AmpR | CEN/ARS | Carvill et al., 2020 |
| Do. R421C | **P**$_{GPD}$-*V5-tef2*-R421C *URA3* AmpR | CEN/ARS | Carvill et al., 2020 |
| pMM09 | *CHP1*$_{L12TAG}$-HA-myc-8×His *URA3* AmpR | CEN/ARS | This work |
| pMM10 | *CHP1*$_{V122TAG}$-HA-myc-8×His *URA3* AmpR | CEN/ARS | This work |
| pMM14 | P $_{T7}$-lacO-6×His-SUMO-Chp1 PT7-Egd1 lacI KanR | *E. coli* | This work |
