# Supplementary material for: Chp1 is a dedicated chaperone at the ribosome that safeguards eEF1A biogenesis
Source: Nat Commun. 2024 Feb 15;15:1382. doi: 10.1038/s41467-024-45645-w (PMC10869706; doi:10.1038/s41467-024-45645-w)
Supplement: Supplementary file 3 — Description of Additional Supplementary Files [file 41467_2024_45645_MOESM3_ESM.pdf]

Description of Additional Supplementary Files

File name: Supplementary Data 1
Description: Chp1 interacting proteins identified by MS.

File name: Supplementary Data 2
Description: ColabFold models (rank 1-5) for the complexes Chp1:Egd2:Egd1 and αNAC:βNAC:PBDC1

File name: Supplementary Data 3
Description: ColabFold models (rank 1-5) for the complex eEF1A domain I:Chp1
