# Supplementary figures and images for: Chp1 is a dedicated chaperone at the ribosome that safeguards eEF1A biogenesis
Source: Nat Commun. 2024 Feb 15;15:1382. doi: 10.1038/s41467-024-45645-w (PMC10869706; doi:10.1038/s41467-024-45645-w)

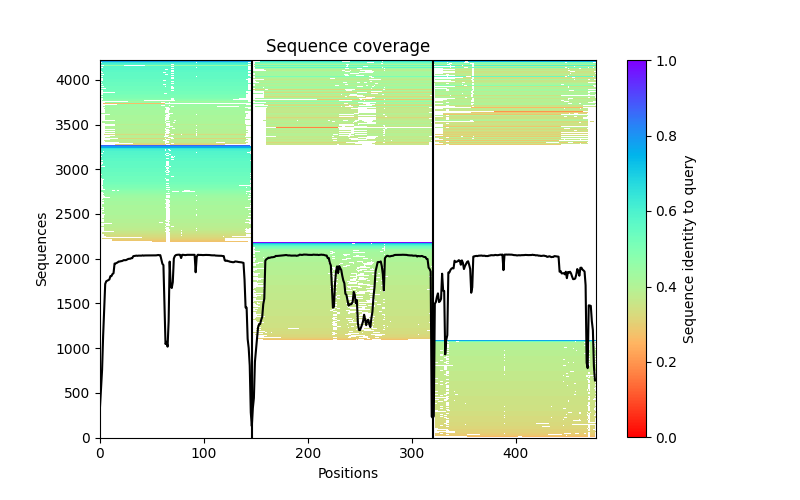

Supplement: Supplementary file 5 — Supplementary Data 2 [file 41467_2024_45645_MOESM5_ESM.zip › Supplmentary Data 2/Chp1_NAC/Chp1Egd2Egd1_bc1be.result/Chp1Egd2Egd1_bc1be_coverage.png]

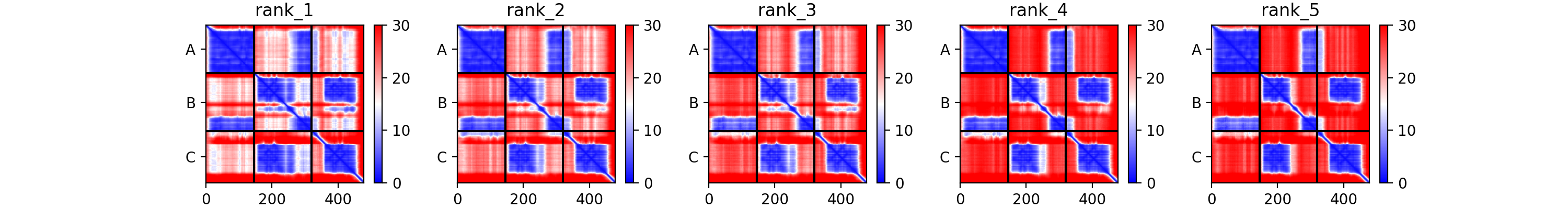

Supplement: Supplementary file 5 — Supplementary Data 2 [file 41467_2024_45645_MOESM5_ESM.zip › Supplmentary Data 2/Chp1_NAC/Chp1Egd2Egd1_bc1be.result/Chp1Egd2Egd1_bc1be_PAE.png]

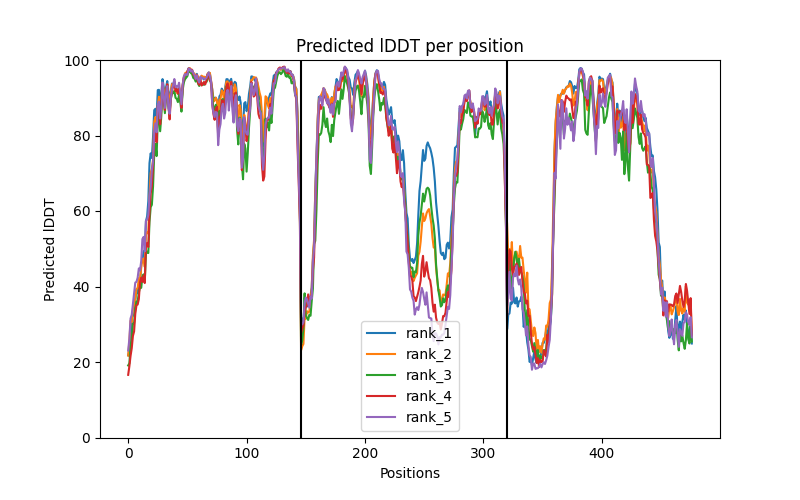

Supplement: Supplementary file 5 — Supplementary Data 2 [file 41467_2024_45645_MOESM5_ESM.zip › Supplmentary Data 2/Chp1_NAC/Chp1Egd2Egd1_bc1be.result/Chp1Egd2Egd1_bc1be_plddt.png]

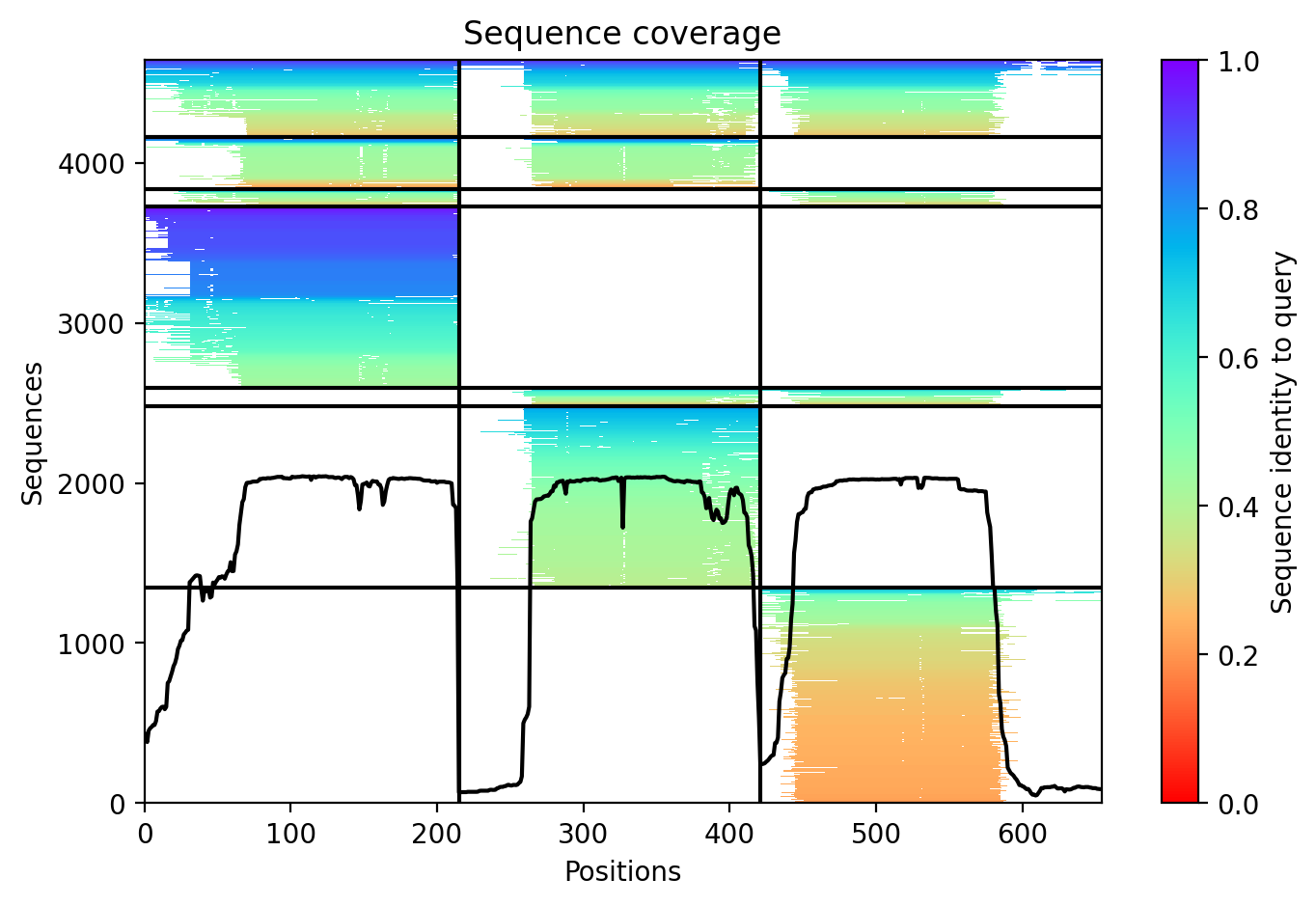

Supplement: Supplementary file 5 — Supplementary Data 2 [file 41467_2024_45645_MOESM5_ESM.zip › Supplmentary Data 2/NAC_PBDC1/NAC_PBDC1_c664a_coverage.png]

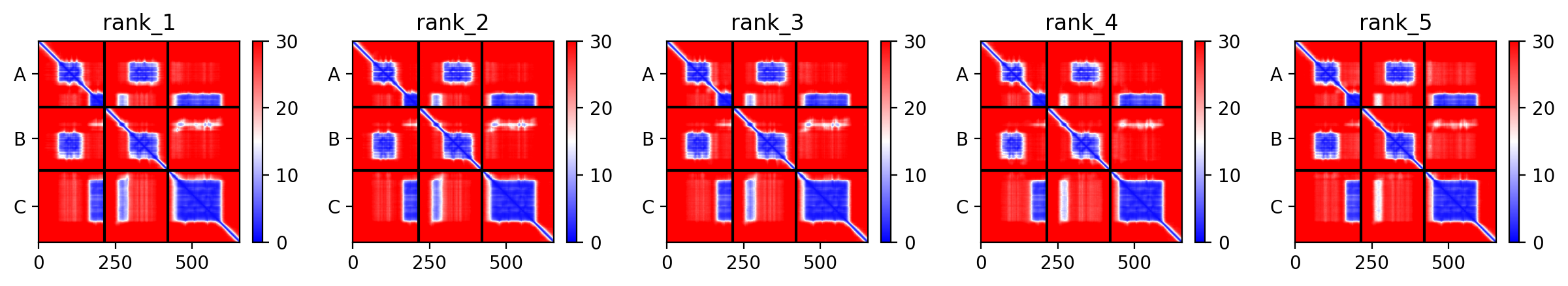

Supplement: Supplementary file 5 — Supplementary Data 2 [file 41467_2024_45645_MOESM5_ESM.zip › Supplmentary Data 2/NAC_PBDC1/NAC_PBDC1_c664a_pae.png]

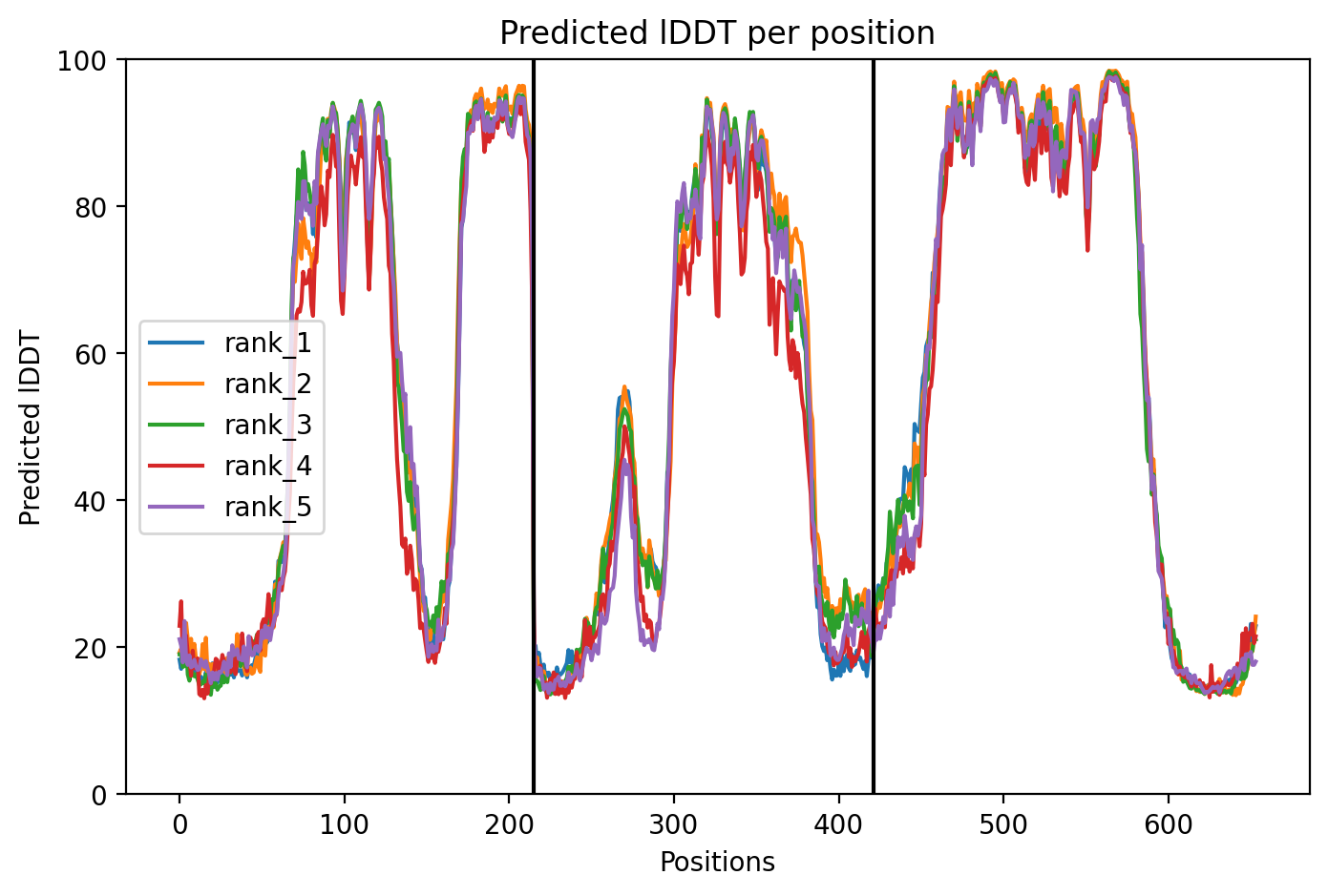

Supplement: Supplementary file 5 — Supplementary Data 2 [file 41467_2024_45645_MOESM5_ESM.zip › Supplmentary Data 2/NAC_PBDC1/NAC_PBDC1_c664a_plddt.png]

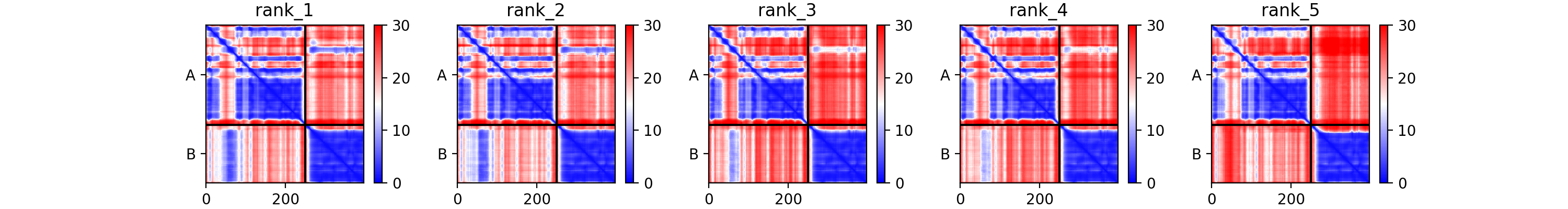

Supplement: Supplementary file 6 — Supplementary Data 3 [file 41467_2024_45645_MOESM6_ESM.zip › TEF1GTPase1250Chp1_5683f.result/TEF1GTPase1250Chp1_5683f_PAE.png]

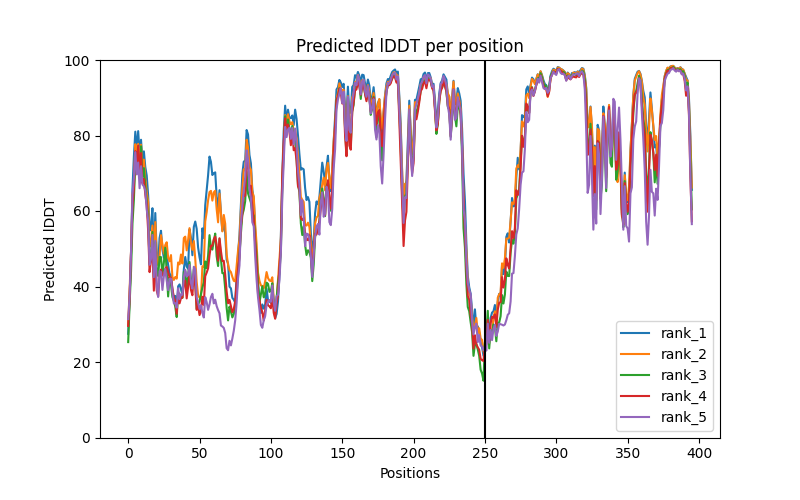

Supplement: Supplementary file 6 — Supplementary Data 3 [file 41467_2024_45645_MOESM6_ESM.zip › TEF1GTPase1250Chp1_5683f.result/TEF1GTPase1250Chp1_5683f_plddt.png]

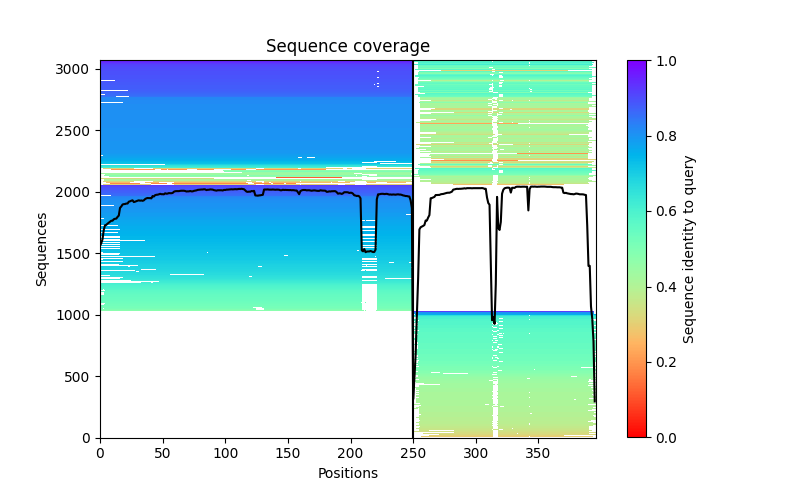

Supplement: Supplementary file 6 — Supplementary Data 3 [file 41467_2024_45645_MOESM6_ESM.zip › TEF1GTPase1250Chp1_5683f.result/TEF1GTPase1250Chp1_5683f_coverage.png]
