# Supplementary material for: Chp1 is a dedicated chaperone at the ribosome that safeguards eEF1A biogenesis
Source: Nat Commun. 2024 Feb 15;15:1382. doi: 10.1038/s41467-024-45645-w (PMC10869706; doi:10.1038/s41467-024-45645-w)
Supplement: Supplementary file 7 — Reporting Summary [file 41467_2024_45645_MOESM7_ESM.pdf]

nature portfolio

Corresponding author(s): Claes Andréasson

Last updated by author(s): 16/1/24

Reporting Summary

Nature Portfolio wishes to improve the reproducibility of the work that we publish. This form provides structure for consistency and transparency in reporting. For further information on Nature Portfolio policies, see our Editorial Policies and the Editorial Policy Checklist.

Statistics

For all statistical analyses, confirm that the following items are present in the figure legend, table legend, main text, or Methods section.

n/a	Confirmed	
☐	☒	The exact sample size (*n*) for each experimental group/condition, given as a discrete number and unit of measurement
☐	☒	A statement on whether measurements were taken from distinct samples or whether the same sample was measured repeatedly
☐	☒	The statistical test(s) used AND whether they are one- or two-sided<br>*Only common tests should be described solely by name; describe more complex techniques in the Methods section.*
☐	☒	A description of all covariates tested
☐	☒	A description of any assumptions or corrections, such as tests of normality and adjustment for multiple comparisons
☐	☒	A full description of the statistical parameters including central tendency (e.g. means) or other basic estimates (e.g. regression coefficient) AND variation (e.g. standard deviation) or associated estimates of uncertainty (e.g. confidence intervals)
☐	☒	For null hypothesis testing, the test statistic (e.g. *F*, *t*, *r*) with confidence intervals, effect sizes, degrees of freedom and *P* value noted<br>*Give P values as exact values whenever suitable.*
☒	☐	For Bayesian analysis, information on the choice of priors and Markov chain Monte Carlo settings
☒	☐	For hierarchical and complex designs, identification of the appropriate level for tests and full reporting of outcomes
☒	☐	Estimates of effect sizes (e.g. Cohen's *d*, Pearson's *r*), indicating how they were calculated

Our web collection on statistics for biologists contains articles on many of the points above.

Software and code

Policy information about availability of computer code

Data collection	No custom code for data collection was used in this study. Software include ImageQuant LAS 4000 (GE Healthcare) imaging system proprietary software, NextSeq control software, Image Lab 5.2.1 software (Biorad), UNICORN 5.11 (GE healthcare), Simplicity 4.2 (Berthold Technologies GmbH, Bad Wildbad, Germany), Image Studio 3.1.4 (LICOR Biosciences), 2300 EnSpire plate reader integrated software, ZEISS ZEN software, Rotor-Gene Q software (Qiagen) and QEx-Orbitrap software (Thermo Finnigan).
Data analysis	Software used for data analysis include Bioconductor package ClusterProfiler (v3.16.1) 42 and DOSE (v3.14.0), DeSeq2, Cutadapt v 3.2, R package RiboSeqTools (DOI https://zenodo.org/records/4016066), custom Julia script (Script 2), Image Lab software (Biorad), Microsoft Office Professional Plus 2016, Image Studio 3.1.4 (LICOR Biosciences), open-source software Fiji, UCSF ChimeraX, ColabFold v1.5.2, Graphpad Prism 7 and 9 and Proteome Discoverer 1.4 Sequest algorithm (Thermo Fisher Scientific).

For manuscripts utilizing custom algorithms or software that are central to the research but not yet described in published literature, software must be made available to editors and reviewers. We strongly encourage code deposition in a community repository (e.g. GitHub). See the Nature Portfolio guidelines for submitting code & software for further information.

Data

Policy information about availability of data

All manuscripts must include a data availability statement. This statement should provide the following information, where applicable:

- Accession codes, unique identifiers, or web links for publicly available datasets
- A description of any restrictions on data availability
- For clinical datasets or third party data, please ensure that the statement adheres to our policy

The sequencing data generated in this study have been deposited in the NCBI's Gene Expression Omnibus60 database under accession code GSE221651 [https://www.ncbi.nlm.nih.gov/geo/query/acc.cgi?acc=GSE221651]. The MS data generated in this study have been deposited to the ProteomeXchange Consortium via the PRIDE61 partner repository under the accession code PXD043391 [http://proteomecentral.proteomexchange.org/cgi/GetDataset?ID=PXD043391]. Source data are provided with this paper.

Research involving human participants, their data, or biological material

Policy information about studies with human participants or human data. See also policy information about sex, gender (identity/presentation), and sexual orientation and race, ethnicity and racism.

Reporting on sex and gender	This study involves mechanistic inquiry regarding fundamental cell biological processes and it is based on using the model organism budding yeast and contains no human data or participants. The data results and interpretation do not have a direct or to our knowledge ,indirect impact on understanding sex and gender nor does sex and gender perspectives change the interpretation of the data.
Reporting on race, ethnicity, or other socially relevant groupings	This study involves mechanistic inquiry regarding fundamental cell biological processes and it is based on using the model organism budding yeast and contains no collection of human data or human participants. The data results and interpretation do not have a direct or to our knowledge, indirect impact on the understanding of race, ethnicity or other socially relevant groupings nor does these categorizations change the interpretation of the data.
Population characteristics	See above.
Recruitment	See above.
Ethics oversight	See above.

Note that full information on the approval of the study protocol must also be provided in the manuscript.

Field-specific reporting

Please select the one below that is the best fit for your research. If you are not sure, read the appropriate sections before making your selection.

☒ Life sciences ☐ Behavioural & social sciences ☐ Ecological, evolutionary & environmental sciences

For a reference copy of the document with all sections, see nature.com/documents/nr-reporting-summary-flat.pdf

Life sciences study design

All studies must disclose on these points even when the disclosure is negative.

Sample size	For each experiment, sample sizes were defined via an n value that represented the number of experimental replicates. The n value is indicated in the respective figure legend. Sample size of the experiments were at least 3, since this is considered sufficient to draw conclusions from costly and highly reproducible biochemical experiments of this type (standard of the field).
Data exclusions	No data were excluded from the analysis.
Replication	To ensure the reproducibility of our data, n experimental replicates were performed as indicated in each respective figure legend. Replicates were fully independent experiments, typically starting with independent cell cultures.
Randomization	Not relevant since the study is purely experimental, and based on genetically defined isogenic yeast strains. Randomization is not critical for this type of experiments since data collection and analysis do not involve subjective assessment. It would not be a rational use of limited resources to implement it.
Blinding	Not relevant since the study is purely experimental, and based on genetically defined isogenic yeast strains. Blinding is not critical for this type of experiments since data collection and analysis do not involve subjective assessment. It would not be a rational use of limited resources to implement it.

Reporting for specific materials, systems and methods

We require information from authors about some types of materials, experimental systems and methods used in many studies. Here, indicate whether each material, system or method listed is relevant to your study. If you are not sure if a list item applies to your research, read the appropriate section before selecting a response.

Materials & experimental systems

n/a	Involved in the study
☐	☒ Antibodies
☐	☒ Eukaryotic cell lines
☒	☐ Palaeontology and archaeology
☒	☐ Animals and other organisms
☒	☐ Clinical data
☒	☐ Dual use research of concern
☒	☐ Plants

Methods

n/a	Involved in the study
☒	☐ ChIP-seq
☒	☐ Flow cytometry
☒	☐ MRI-based neuroimaging

Antibodies

Antibodies used

Used antibodies, dilution and reference: α-eEF1A rabbit 1:10000 (ED7001; Kerafast, inc.), α-Fes1 rabbit 1:5000 (Gowda et al. 2016, Mol Biol Cell), α-Btn2 rabbit 1:5000 (Miller et al. 2015 EMBO J), α-Pgk1 22C5D8 mouse 1:5000 (459250; Thermo Fisher Scientific), anti-NAC 1:5000 rabbit (Koplin et al. 2010 JCB); anti-HA 3F10 Rat 1:5000 (11867423001 Roche product line, Merck KgaA), anti-GFP 7.1/13.1 mouse 1:5000 (11814460001, Roche product line, Merck KgaA), anti-Chp1 1:1000 rabbit (this study), anti-V5 Sv5-Pk1 mouse 1:5000 (R960-25; Thermo Fisher Scientific), anti-GAPDH 1D4 mouse 1:5000 (MA1-16757; Thermo Fisher Scientific), anti-ubiquitin HRP P4D1 mouse 1:1000 (sc-526508; Santa Cruz Biotechnology, Inc), anti-6X His tag HIS.H8 mouse 1:5000 (ab18184; Abcam plc.), anti-Rpl8 (recognizes yeast Rpl2) rabbit 1:1000 (PA5-41713; Thermo Fisher Scientific), anti-Rpl25 rabbit 1:5000 (Zhang et al. 2012 Mol Bio Cell), anti-Egd2 rabbit 1:2000 (this study), anti-Hsp42 rabbit 1:5000 (Ho et al. 2019 Nat. Commun.), anti-FLAG M2 mouse 1:10000 (F1804; Merck KGaA), anti-Myc peroxidase 9E10 1:5000 (11814150001; Roche product line, Merck KgaA).

Validation

α-eEF1A rabbit 1:10000 (ED7001; manufacturer's validation Kerafast, inc., recognized Tef1/2 in yeast), α-Fes1 rabbit 1:5000 (validated in Gowda et al. 2016, Mol Biol Cell), α-Btn2 rabbit 1:5000 (validated in Miller et al. 2015 EMBO J), α-Pgk1 22C5D8 mouse 1:5000 (459250; manufacturer's validation Thermo Fisher Scientific, recognizes Pgk1 in yeast), anti-NAC 1:5000 rabbit (validated in Koplin et al. 2010 JCB); anti-HA 3F10 Rat 1:5000 (11867423001 Roche product line, manufacturer's validation Merck KgaA), anti-GFP 7.1/13.1 mouse 1:5000 (11814460001, Roche product line, manufacturer's validation Merck KgaA), anti-Chp1 1:1000 rabbit (this study), anti-V5 Sv5-Pk1 mouse 1:5000 (R960-25; manufacturer's validation Thermo Fisher Scientific), anti-GAPDH 1D4 mouse 1:5000 (MA1-16757; manufacturer's validation Thermo Fisher Scientific, recognizes Tdh1 in yeast), anti-ubiquitin HRP P4D1 mouse 1:1000 (sc-526508; manufacturer's validation Santa Cruz Biotechnology, Inc), anti-6X His tag HIS.H8 mouse 1:5000 (ab18184; manufacturer's validation Abcam plc.), anti-Rpl8 (recognizes yeast Rpl2) rabbit 1:1000 (PA5-41713; manufacturer's validation Thermo Fisher Scientific), anti-Rpl25 rabbit 1:5000 (validated in Zhang et al. 2012 Mol Bio Cell), anti-Egd2 rabbit 1:2000 (this study), anti-Hsp42 rabbit 1:5000 (validated in Ho et al. 2019 Nat. Commun.), anti-FLAG M2 mouse 1:10000 (F1804; manufacturer's validation Merck KGaA), anti-Myc peroxidase 9E10 1:5000 (11814150001; Roche product line, manufacturer's validation Merck KgaA).

Eukaryotic cell lines

Policy information about cell lines and Sex and Gender in Research

Cell line source(s)	No cell lines were used in the study.
Authentication	See above.
Mycoplasma contamination	See above.
Commonly misidentified lines (See ICLAC register)	See above.
